# Comparing the quantity and quality of randomised placebo-controlled trials of antibiotics for acute respiratory, urinary, and skin and soft tissue infections: a scoping review

**DOI:** 10.3399/bjgpopen20X101082

**Published:** 2020-09-30

**Authors:** Mina Bakhit, Tammy Hoffmann, Miriam Santer, Matthew Ridd, Nick Francis, Eva Hummers, Justin Clark, Carmen Hilliges, Chris Del Mar

**Affiliations:** 1 Institute for Evidence-Based Healthcare, Bond University, Gold Coast, Queensland, Australia; 2 Primary Care, Population Sciences and Medical Education, University of Southampton, Southampton, UK; 3 Population Health Sciences, University of Bristol, Bristol, UK; 4 Department of General Practice, Göttingen University Medical Centre, Göttingen, Germany

**Keywords:** anti-bacterial agent, primary health care, respiratory tract infections, urinary tract infections, skin and soft tissues infections, randomised controlled trials

## Abstract

**Background:**

The management of acute respiratory infections (ARIs), urinary tract infections (UTIs), and skin and soft tissue infections (SSTIs) should be guided by high quality evidence.

**Aim:**

To compare the quantity and quality of randomised placebo-controlled trials of antibiotics for ARIs, UTIs, and SSTIs.

**Design & setting:**

A scoping review of the literature was performed using comprehensive search strategies.

**Method:**

PubMed and the Cochrane Central Register of Controlled Trials (CENTRAL) were searched for published studies from inception until 17 April 2019. Randomised controlled trials (RCTs) that compared participants in primary care or in the community who had uncomplicated acute ARI, UTI, or studies, and were randomised to antibiotic or placebo (or no active treatment), were eligible for inclusion. Two groups of researchers independently screened articles for inclusion, extracted data, and assessed the quality of included studies.

**Results:**

A total of 108 eligible studies were identified: 80 on ARI, eight on UTI, and 20 on SSTI. The quality of studies varied with unclear risk of bias (RoB) prevalent in many domains. There was a gradual improvement in the quality of trials investigating ARIs over time, which could not be assessed in SSTI and UTI studies.

**Conclusion:**

This review highlights a sparsity of trials assessing the effectiveness of antibiotics in people with UTIs and SSTIs, compared to trials targeting ARIs. This gap in the evidence needs to be addressed by conducting further high quality trials on the effects of antibiotics in patients with UTI and SSTI.

## How this fits in

There is a sparsity of high quality evidence on the effectiveness of antibiotics for UTIs and SSTIs, which makes it difficult to produce evidence-based guidelines and patient decision aids for these conditions. This scoping review highlights an important gap in the evidence that needs to be addressed by conducting further high quality trials in patients with UTI and SSTI.

## Introduction

Widespread antibiotic resistance now threatens a post-antibiotic era.^1,2^ Reducing antibiotic use is widely recognised as a key component to tackling the global antibiotic resistance crisis. Primary care is an important focus for this strategy as this is where most antibiotics are prescribed and where the benefits from use of antibiotics are often limited.^3,4^ To assist clinicians in prescribing antibiotics appropriately, knowledge about the benefits and harms of their use in various clinical situations is needed.

ARIs, SSTIs, and uncomplicated UTIs are the three most common infections seen in primary care, with ARIs accounting for 41%–46%, SSTIs accounting for 16%–18%, and UTIs accounting for 9%–23% of antibiotics prescribed outside hospitals.^5,6^ However, an apparent lack of published scientific studies on the effectiveness of antibiotics for treating SSTIs and UTIs compared with ARIs was identified. A lack of high quality evidence on the effectiveness of antibiotics for SSTIs and UTIs makes it difficult to produce evidence-based guidelines and patient decision aids for these conditions. There are also implications for a primary research agenda. Accordingly, the authors decided to explore this impression by conducting a scoping review of the literature on studies of the effectiveness of antibiotics versus placebo (or no treatment) for these infections, and compare the quantity and quality of the RCTs addressing this question for these groups of infections.

## Method

### Eligibility criteria

RCTs were included that compared antibiotic-exposed participants (alone or with any other concomitant treatment if that was also available to the placebo group) to placebo (or no treatment) controls among patients managed in primary care or in the community, who had uncomplicated acute infections of either the respiratory tract, urinary tract, or skin and soft tissue. Studies were excluded that: 1) did not have a placebo or no-treatment control; 2) focused on chronic or complicated acute infections (for example, persistent cough following acute bronchiolitis, chronic obstructive pulmonary disease [COPD], and/or infected diabetic foot ulcers); 3) had recruited hospitalised participants; and 4) had recruited emergency department patients (except for studies conducted in countries or parts of countries where they do not have family or general practices like some states in the US).

### Information sources and search strategy

PubMed and CENTRAL were searched from inception until 17 April 2019. Searches for all three conditions were done on the same day using the same methods, other than a change of condition-specific search terms (see Supplementary Appendix S1 for full search strategy). After identifying the included studies, a forward and backward citation analysis was performed using the Scopus database. To facilitate the screening process, RobotSearch engine was used to exclude all obvious non-RCTs. RobotSearch engine is a validated search engine with high sensitivity and specificity.^7^

### Study selection

Two groups of researchers independently screened the pool of potentially eligible studies using Endnote X9 software or the Rayyan website.^8^ A third reviewer resolved any disagreement.

### Data extraction and RoB assessment

A data extraction form was drafted by the research team and the topic areas were divided up between the authors (respiratory, urinary, and skin and soft tissue). Each group of authors trialled the form on a handful of their different study types and suggested changes. The finalised form was then applied by each team across all study types (See Supplementary Box S1 for data extraction form). Two groups of authors used a data extraction form to independently extract data on the studied condition (and diagnosis), country, sample size, and RoB using items from the Cochrane Risk of Bias tool for RCTs.^9^

### Synthesis of results

The data were synthesised descriptively, presenting counts, summary tables, and figures that describe the number, type, and quality of studies identified.

## Results

**Figure 1.**
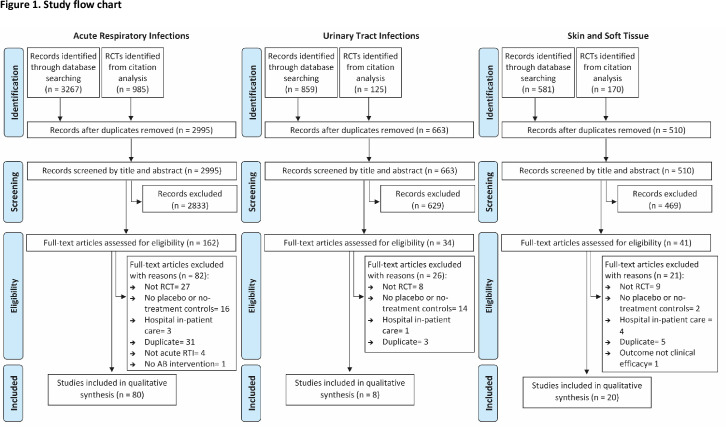
Study flow chart. AB = Antibiotics, RCT = randomised controlled trial, RTI = respiratory tract infection

Figure 1 shows the flow of articles throughout the study. A total of 108 studies were included in the final descriptive synthesis. Most studies addressed ARI (*n* = 80), followed by SSTI (*n* = 20), and then UTI (*n* = 8) (see Supplementary Box S2 for a complete list of included RCTs).

### Quantity of RCTs over time for each of the conditions

**Figure 2.**
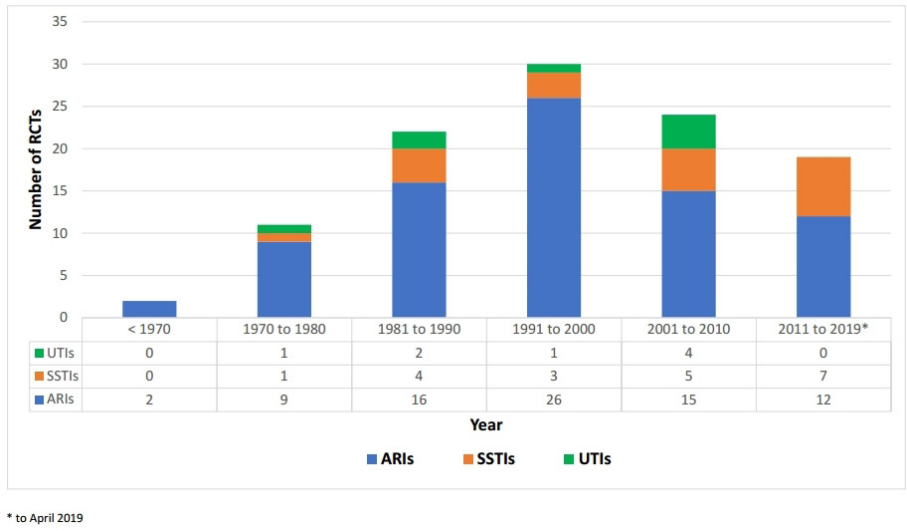
Number of randomised controlled trials (RCTs) published every decade for acute respiratory infections (ARIs), skin and soft tissue infections (SSTIs), and urinary tract infections (UTIs)

The earliest published eligible trial identified was published in 1956, and there was a gradual increase in the number of trials published each decade, with a peak for the decade 1991–2000, which saw 30 RCTs published (28% of total) (Figure 2). The number of RCTs about antibiotic effectiveness for SSTIs increased during the period of 2001 to the date of the search (17 April 2019). However, for UTI, there were no new RCTs from 2011 until 2019.

### Study characteristics

Within each condition, the RCTs investigated a range of diagnoses. The most common are highlighted below (refer to Supplementary Table S1 for the characteristics of each RCT). All but 10 RCTs were conducted in one of the Organisation for Economic Cooperation and Development countries (OECD). Of the ARI RCTs, 19 investigated sinusitis, 17 sore throat, 14 otitis media, 13 non-specific ARI, 12 bronchitis, two non-severe pneumonia, two laryngitis, and one rhinitis. Of the SSTI RCTs, six investigated skin abscess, four impetigo, three atopic dermatitis, three non-specific SSTI, two erythrasma, one skin sores, and one infected traumatic lesion. Of the UTI RCTs, seven investigated non-specific UTI and one cystitis.

### Quality of RCTs over time for each of the conditions

**Figure 3.**
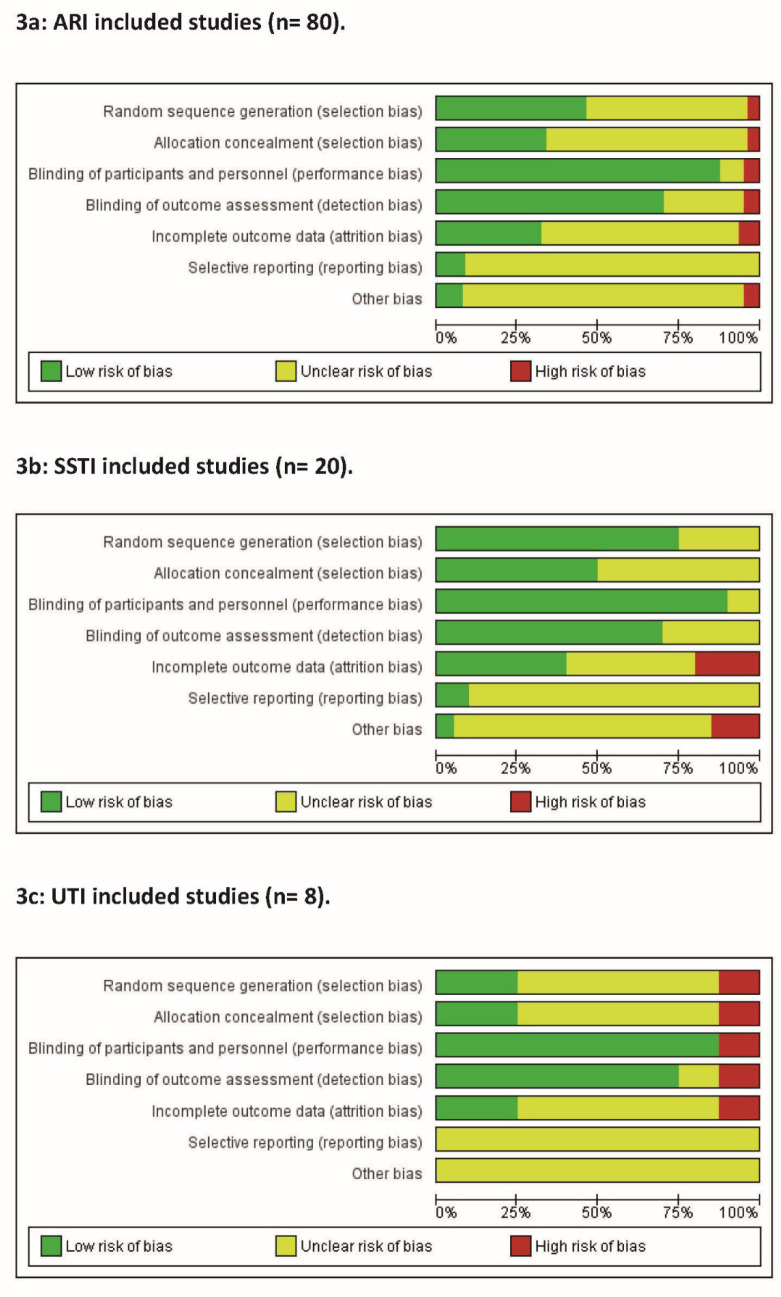
Risk of bias graph: review authors' judgements about each risk of bias item presented as percentages across all included studies. ARI = acute respiratory infection. SSTI = skin and soft tissue infection. UTI = urinary tract infection

Of the 80 ARI trials, a minimal number of trials were at ‘high RoB' across all domains. On the other hand, ‘unclear RoB’ was more common across most domains (Figure 3a). The methodological quality of ARI studies improved over time. See Supplementary Figure S1 for RoB assessment for individual studies ordered according to year of publishing.

Of the 20 SSTI trials, a ‘high RoB’ was detected in only two of the domains but an ‘unclear RoB’ was more common across most domains (Figure 3b). See Supplementary Figure S2 for RoB assessment for individual studies.

Across the eight UTI trials, a ‘high RoB’ occurred in five of the domains, with ‘unclear RoB’ common across most domains (Figure 3c). See Supplementary Figure S3 for RoB assessment for individual studies.

## Discussion

### Summary

In this rigorous scoping review of RCTs comparing antibiotic treatment with no active treatment for common infections, a marked difference was found in the number of trials addressing ARIs (*n* = 80), SSTIs (*n* = 20), and UTIs (*n* = 8). The quality of the reported studies was mixed for all three groups of infections, with many studies having an unclear or high RoB in many domains.

Reasons for the noticeable differences in the number of studies is unclear. ARIs are more common than SSTIs and UTIs, and account for more antibiotic use, but even if this is taken into account, the number of trials in ARIs relative to the incidence or prevalence of the condition is still much greater than for SSTIs or UTIs. One might argue that it is misleading to consider this by condition and it may make more sense to consider the number of studies by individual diagnosis. One of the reasons why these infections are often classified by system is that there is overlap between individual ‘diseases’ (for example, rhinitis, sinusitis, rhinosinusitis, common cold, sore throat, pharyngitis, and tonsillitis). Nevertheless, the number of studies was looked at by ‘diagnosis’ and still more were found for ARIs (19 for sinusitis, 17 for sore throat, and 14 for otitis media) compared with SSTIs (six for skin abscess and four for impetigo) or UTIs (seven for non-specific UTI and one for cystitis).

The quality of RCTs has generally improved over time. Therefore, an argument might be made that many of the trials of antibiotics for ARI trials were conducted many decades ago when RCT standards were lower, without the availability of a reporting guideline for RCTs (for example, the Consolidated Standards of Reporting Trials [CONSORT] statement that was published in 2001), and therefore needed repeating more recently, whereas this was not the case for most of the SSTI and UTI trials, which were high quality trials conducted more recently. However, although most of the ARI trials were conducted prior to 2000, there has been a continuing of more ARI trials than SSTI or UTI trials in more recent years. Furthermore, the overall quality of the trials is similar for the three groups of infections.

A gradual increase in the number of SSTI trials conducted over time was found, suggesting that there has been an increase in interest about the effectiveness of antibiotics for SSTIs. However, although there was an increase in the number of UTI trials in 2001–2010 relative to the decades before, no trials were found on the effectiveness of antibiotics published since 2010. Another possible explanation for the findings could be that despite there being a relatively small number of trials conducted for SSTIs and UTIs they were nevertheless able to produce more certain results such that further trials were not indicated, whereas for ARIs the effect sizes have been smaller and/or the results less conclusive, resulting in the need for further studies. However, this argument is not supported as the body of evidence does not provide definitive answers about the effects of antibiotic treatment for SSTIs and UTIs. For example, the available evidence suggests that oral antibiotics are inferior to topical antibiotics for localised impetigo and there are insufficient data on their effectiveness for non-localised impetigo,^10^ yet they are frequently used for all forms of impetigo. For UTI, there is evidence from a meta-analysis that antibiotics increase the chance of cure or improvement compared with placebo in women with uncomplicated UTI.^11,12^ However, there is a reasonably high spontaneous cure rate,^13^ complications are rare, adverse effects are relatively frequent, and there is uncertainty in relation to how the effectiveness varies by baseline symptoms and risk factors.

### Strengths and limitations

Comprehensive search strategies were used for this scoping review. It had at least two reviewers who independently decided on the eligibility of each paper, with a third reviewer as an arbiter. Independent assessments were conducted of the RoB for each RCT. The purpose of this review was to identify and compare the quantity and quality of research evidence for the three groups of common infections rather than establish a pooled estimate of effect. It could be argued that the number of studies is not important, and that recommendations should consider the effect sizes and confidence intervals found in the available studies, and possibly the overall number of patients included. Nevertheless, as discussed above, uncertainty regarding the use of antibiotics remains for many SSTI and UTI scenarios. A potential limitation is that only RCTs with placebo or no active treatment as a comparator were included. However, for conditions where there are strong clinical beliefs about the effectiveness of antibiotics, such as cellulitis and UTI, it may be difficult to conduct trials with no active comparator and studies comparing antibiotics with other treatments, such as analgesics, may be appropriate.

### Comparison with existing literature

To the authors’ knowledge, no other studies have directly compared the quantity and quality of RCTs assessing the effects of antibiotics for ARIs, UTIs, and SSTIs. In a study looking at the extent to which recommendations for primary care practice are guided by high quality evidence within the different clinical areas,^14^ Ebell *et al* found that only 18% of the recommendations for primary care are based on evidence from consistent high quality studies and some clinical areas were most investigated with the highest grade A (using the Strength of Recommendations Taxonomy [SORT]^15^) clinical recommendations: such as pregnancy and childcare, cardiovascular, and other areas with the least type of recommendations such as musculoskeletal, rheumatological, and haematological.

### Implications for research and practice

This scoping review has highlighted a sparsity of trials assessing the effects of antibiotics in people with SSTIs and UTIs, relative to the number of trials assessing this question for ARIs. This suggests an important gap in the evidence that needs to be addressed by conducting further high quality trials in patients with SSTIs and UTIs. Clinicians have to contend with greater uncertainty about the benefits and harms of interventions (in this case, antibiotics for SSTIs and UTIs), which are underpinned by much less high quality empirical evidence, denoting a weaker recommendation. Communication of the ambiguity of evidence is vital to allow patients to make an informed decision regarding their treatment option. Resistance to antibiotics that are typically used to treat SSTI, such as flucloxacillin, and UTI, such as trimethoprim, is common and problematic; therefore, it is particularly important to address antimicrobial stewardship in these areas. The first step in doing so is to generate robust evidence regarding the benefits and harms of using antibiotics to treat these conditions.
